# Increased resistance towards fatigability in patients with facioscapulohumeral muscular dystrophy

**DOI:** 10.1007/s00421-021-04650-3

**Published:** 2021-03-01

**Authors:** Matteo Beretta-Piccoli, Luca Calanni, Massimo Negro, Giulia Ricci, Cinzia Bettio, Marco Barbero, Angela Berardinelli, Gabriele Siciliano, Rossella Tupler, Emiliano Soldini, Corrado Cescon, Giuseppe D’Antona

**Affiliations:** 1 Criams-Sport Medicine Centre Voghera, University of Pavia, Voghera, Italy; 2 Rehabilitation Research Laboratory 2rLab, Department of Business Economics, Health and Social Care, University of Applied Sciences and Arts of Southern Switzerland, Manno/Landquart, Switzerland; 3 Department of Clinical and Experimental Medicine, University of Pisa, Pisa, Italy; 4 Department of Life Sciences, University of Modena and Reggio Emilia, Modena, Italy; 5 Child Neuropsychiatry, IRCCS Mondino Foundation, Pavia, Italy; 6 Research Methodology Competence Centre, Department of Business Economics, Health and Social Care, University of Applied Sciences and Arts of Southern Switzerland, Manno, Switzerland; 7 Department of Public Health, Experimental and Forensic Medicine and Sport Medicine Centre Voghera, University of Pavia, via Foscolo, 13 – 27058 Voghera, Italy

**Keywords:** Neuromuscular disease, Dystrophy, Electromyography, Fatigability, Biceps brachii

## Abstract

**Purpose:**

In facioscapulohumeral muscular dystrophy (FSHD) fatigue is a major complaint. We aimed to investigate whether during isometric sustained elbow flexions, performance fatigability indexes differ in patients with FSHD with respect to healthy controls.

**Methods:**

Seventeen patients with FSHD and seventeen healthy controls performed two isometric flexions of the dominant biceps brachii at 20% of their maximal voluntary contraction (MVC) for 2 min and then at 60% MVC until exhaustion. Muscle weakness was characterized as a percentage of predicted values. Maximal voluntary strength, endurance time and performance fatigability indices (mean frequency of the power spectrum (MNF), muscle fiber conduction velocity (CV) and fractal dimension (FD)), extracted from the surface electromyogram signal (sEMG) were compared between the two groups.

**Results:**

In patients with FSHD, maximal voluntary strength was 68.7% of predicted value (*p* < 0.01). Compared to healthy controls, FSHD patients showed reduced MVC (*p* < 0.001; *r* = 0.62) and lower levels of performance fatigability, characterized by reduced rate of changes in MNF (*p* < 0.01; *r* = 0.56), CV (*p* < 0.05; 0.37) and FD (*p* < 0.001; *r* = 0.51) and increased endurance time (*p* < 0.001; *r* = 0.63), during the isometric contraction at 60% MVC.

**Conclusion:**

A decreased reduction in the slopes of all the considered sEMG parameters during sustained isometric elbow flexions suggests that patients with FSHD experience lower levels of performance fatigability compared to healthy controls.

## Introduction

Fatigue is known to be a common symptom in muscular dystrophies (Kalkman et al. 2005). Among the dystrophies whose genetic defects have been identified at the molecular level, facioscapulohumeral muscular dystrophy (FSHD), presents the most peculiar mutation: the FSHD gene defect is in fact not located in any protein-coding gene (Tupler and Gabellini 2004). Instead, in the majority of patients, the disease was associated with the contraction of a polymorphic region known as D4Z4 (chromosome 4q35) that is characterized by an array of tandemly repeated units of 3.3 kb (van Deutekom et al. 1993; Wijmenga et al. 1992). The primary mediator of FSHD pathology is thought to be the expression of DUX4, a gene epigenetically repressed in most somatic tissues located in each unit of the D4Z4 repeat array (Lemmers et al. 2010). In patients with FSHD, inadequate DUX4 protein expression in skeletal muscle is favored by D4Z4 chromatin relaxation (Lassche et al. 2020). Clinically, FSHD is characterized by slowly progressive weakness of the muscles of the face, shoulder, and upper arm. However, many patients also have weakness of the trunk and leg muscles; in fact, sometimes these are the most pronounced symptoms. In addition, some patients have no symptoms or only very mild symptoms (Mul et al. 2016). Progression of the disease is associated with atrophy and fatty infiltration of the muscle tissue which can be visualized on MRI (Mul et al. 2017).

In FSHD, fatigue occurs as an early leitmotif of the disease and as a disabling symptom in daily activities; in a recent qualitative study, patients described fatigue as “an overwhelming and unpredictable experience” without recognizing the underlying causes, making it difficult to cope with and thus massively affecting participation, social contacts, and quality of life (Schipper et al. 2017). Furthermore, in a survey involving 328 participants with FSHD, one of the symptoms with the highest prevalence was fatigue (93.8%) (Hamel et al. 2019).

For the purposes of this study, fatigue will be discussed within the taxonomy proposed by Kluger et al. (2013). Specifically, fatigue is defined as a symptom or perception, characterized by feelings of tiredness and weakness, in which physical and cognitive functions are impaired because of interactions between performance fatigability and perceived fatigability. Performance fatigability refers to the decline in an objective measure of performance, such as the generation of maximal voluntary force (MVC), the ability to provide an adequate signal for voluntary muscle activation, or the involuntary twitch response to stimulation (Enoka and Duchateau 2016). To assess muscle contractility or the degree of muscle activation before and during a performance task in FSHD, several studies have been conducted using electrophysiological responses evoked by muscle or peripheral nerve stimulation. For example, Schulte-Mattler et al. (2003) described excessive fatigability during isometric ankle dorsiflexion in four patients with FSHD. Later, Schillings et al. (2007) described reduced performance fatigability after a 2-min sustained isometric MVC of the biceps brachii (BB) in 65 patients with FSHD, compared to control subjects. Recently, Bachasson et al. (2014) demonstrated similar fatigability during intermittent isometric contractions in the quadriceps femoris in 19 FSHD patients compared to controls, using femoral nerve magnetic stimulation. However, to overcome the limitations of electrical/magnetic stimulation in clinical populations (discussed in Place and Millet 2020), such as the impossibility to test neuromuscular function under physiological conditions, pain during stimulation that can lead to biased measurements, the contribution of intramuscular processes to the superimposed force during fatigue and insufficient stimulation intensity, performance fatigability can be assessed using surface electromyography (sEMG). For example, spectral parameters, muscle fiber conduction velocity (CV) or non-linear parameters (reviewed in Rampichini et al. 2020) are suitable to be used as indirect indices of performance fatigability. Indeed, fatigability during isometric constant force contractions, can be observed by the decrease of CV mainly related to a decrease of intracellular pH (Komi and Tesch 1979). A decrease in the fractal dimension (FD) of the sEMG signal has been associated with fatigability, aging, and disease (Beretta-Piccoli et al. 2020; Goldberger et al. 2002; Arjunan and Kumar 2013). These findings suggest a potential utility of the fractal analysis of the sEMG signal as a complementary tool for assessing fatigability during a performance test.

Therefore, the primary objective of this study was to investigate whether patients fatigue differently than healthy controls (HC) during sustained isometric elbow flexions. We hypothesized that since FSHD is characterized by a transition from fast-glycolytic to slow-oxidative muscle fibres (Celegato et al. 2006), patients would show less performance fatigability compared to controls.

## Methods

### Participants

This cross-sectional study was performed according to the Declaration of Helsinki, with the approval of the local Ethics Committee of the University of Pisa. Seventeen patients with FSHD and seventeen HC were recruited after providing written informed consent.

The study was part of a crowdfunding project (#Sport4therapy) conducted at CRIAMS-Sport Medicine Centre Voghera and sponsored by the University of Pavia, aiming to identify the correct sports therapy approach in patients affected by rare neuromuscular diseases (Siciliano et al. 2019), including FSHD (Berardinelli and D'Antona 2019). Data collection began in 2013 and was completed in 2019. Inclusion criteria were age of ≥ 16 years, a clinical or genetic diagnosis of FSHD, and enrollment in the Italian National Registry for FSHD. Exclusion criteria were wheelchair bound at selection, use of corticosteroids, severe cardiac and respiratory dysfunction, and psychological/psychiatric disorders. A diagnosis of FSHD had to be confirmed by DNA testing (Lemmers et al. 2012) at the University of Modena and Reggio Emilia (Italy). Patients with FSHD were enrolled in the study. The severity of the disease was assessed using the FSHD clinical score (Lamperti et al. 2010), which ranges from 0, when there are no objective signs of functional impairment, to 15, when all tested muscle groups are severely impaired and the patient is wheelchair dependent. Each section describes the functional assessment of six muscle districts that are peculiarly affected in FSHD: face (score 0–2); shoulder girdle (score 0–3); upper limbs (score 0–2); distal legs (score 0–2); pelvic girdle (score 0–5); abdominal muscles (score 0–1). The protocol assigns an independent score to each muscle group, providing an accurate description of the distribution of muscle weakness for each individual. The main characteristics of the patients are listed in Table 1.

**Table 1:** Descriptive statistics of the socio-demographic and clinical variables

	*n*	Median	IQR
*Socio-demographic variables*			
Gender			
Woman	8	–	–
Man	9	–	–
Age	–	33.0	31.25
*Clinical variables*			
FSHD categories			
A	12	–	–
B	3	–	–
C	0		
D	2	–	–
FSHD asymmetry			
Right > Left	9	–	–
Right = Left	5	–	–
Right < Left	3	–	–
D4Z4 contraction (kb)/number of alleles	–	27.00	11.50
(11–19)/ 1–3	2		
(20–26)/ 4–5	7		
(27–31)/ 6	3		
(33–35)/ 7–8	3		
(36–41)/ 9–10	2		
D4Z4 contraction (kb)	–	27.00	11.50
Checklist individual strength^a^	–	25.50	15.00
Severity of FSHD (clinical score)	–	4.00	6.25
Scapular girdle involvement score		2.00	1.00

^a^Variable with three missing values (n = 14)

*FSHD* facioscapulohumeral muscular dystrophy, *IQR* interquartile range

Categories definition see text

### FSHD categories

Patients were allocated to the four clinical categories according to the Comprehensive Clinical Evaluation Form (CCEF, Ricci et al. 2016). The CCEF classifies (1) subjects with facial and scapular girdle muscle weakness typical of FSHD (category A), (2) subjects with muscle weakness limited to the scapular girdle or facial muscles (category B), (3) asymptomatic/healthy subjects (category C), and (4) subjects with a myopathic phenotype presenting clinical features not consistent with the canonical phenotype of FSHD (category D).

Since patients belonging to category A have the most peculiar signs of the disease, they were compared with the other categories, to highlight differences in the clinical variables considered.

### FSHD asymmetry

Significant asymmetry of muscle involvement was previously observed in the upper extremities, showing right-sided dominance, regardless of handedness (Rijken et al. 2014). Asymmetry of muscle involvement was clinically evaluated and a comparative analysis between patients with predominant right-sided or left-sided involvement was performed to determine if side involvement correlated with the clinical severity of the disease.

### Experimental procedures

#### Perceived fatigability

The degree of perceived fatigability was assessed before the fatigue task using the fatigue subscale of the Checklist Individual Strength (CIS fatigue). This scale consists of eight questions regarding fatigability experienced during the previous 2 weeks; each question was scored on a 7-point Likert scale (Vercoulen et al. 1994). A total score ≥ 35 indicates severe fatigue. The CIS fatigue has good internal consistency (Cronbach a 0.83–0.92), high discriminant validity, and high sensitivity to change in patients with FSHD (Kalkman et al. 2007).

#### Performance fatigability

The selected protocol has been shown to induce fatigue in the elbow flexors in healthy subjects and patients (Beretta-Piccoli et al. 2017, 2020). Briefly, participants were asked to perform two MVCs, separated by 2 min rest, followed by a 20% MVC contraction that lasted 2 min and a 60% MVC that was held until the force level dropped below 90% of the target (endurance time, i.e. the time for which a subject can sustain the requested mechanical task). The two sub-maximal contractions were separated by a 5-min rest. Since arm muscles show early disability in FSHD (Derry et al. 2012; Rijken et al. 2014; Tawil 2018), the BB was selected as the affected muscle. In particular, the BB is subject to selective muscle wasting resulting in so-called ‘Popeye’ arms because of the contrast between atrophied perihumeral musculature and sparing of the forearm and distal deltoid muscles (Mul et al. 2016). Furthermore, because this muscle has long fibers that run parallel to the skin, high quality sEMG acquisitions, according to Barbero et al. (2012) may be recorded. Finally, sub-maximal contractions were selected because they are more representative of the intensity of activities performed during daily life.

#### EMG and force measurements

Myoelectric signals were recorded from the dominant BB in a monopolar configuration. Participants were seated on a height-adjustable chair with their arm positioned on an isometric ergometer (MUC1, OT-Bioelettronica, Turin, Italy), equipped with a load cell (Model TF022, CCT Transducers, Turin, Italy). The wrist was fastened to the ergometer, with the elbow flexed at 120°. A bi-dimensional array of 64 electrodes (3 mm diameter, 8 × 8 grid, 10 mm interelectrode spacing; model ELSCH064NM3; OT-Bioelettronica) was positioned on the BB according to Barbero et al. (2012) with the distal edge close to the cubital fossa and the midline of the array aligned with the midline of the BB along a line from the cubital fossa to the acromion. The ground electrode was placed on the contralateral wrist.

The torque of the elbow was measured using a torque meter operating linearly in the range 0–1000 Nm. The torque signal was amplified (MISO II; OT-Bioelettronica) and saved on a computer. The EMG signals were amplified with a variable factor ranging from 2000 to 5000 (EMG-USB2 + ; OT-Bioelettronica), filtered with the hardware filter (10–500 Hz bandpass) and then with an offline Butterworth anti-causal bandpass filter (2nd order—20–400 Hz bandpass) and sampled together with the torque signal at 2048 Hz using a 16-bit A/D converter, with 5 V dynamic range, and stored on a computer. The torque signal was displayed on a screen, as real-time biofeedback.

#### Assessment of muscle weakness

Normative data on the maximal voluntary isometric force exerted by healthy subjects at 90° elbow flexion were retrieved from the study by Meldrum et al. (2007). The value was then corrected for a 60° elbow flexion using the following predictive equation from Bober et al. (2002):

$$Y\left( \% \right) \, = { 55}.{49 } + \, 0.{88}x + \, 0.00{4}x^{{2}} {-} \, 0.000{1}x^{{3}} ,$$

where *Y* is the estimated peak torque, and *x* is the specific joint angle.

#### Signal processing

**Fig. 1:**
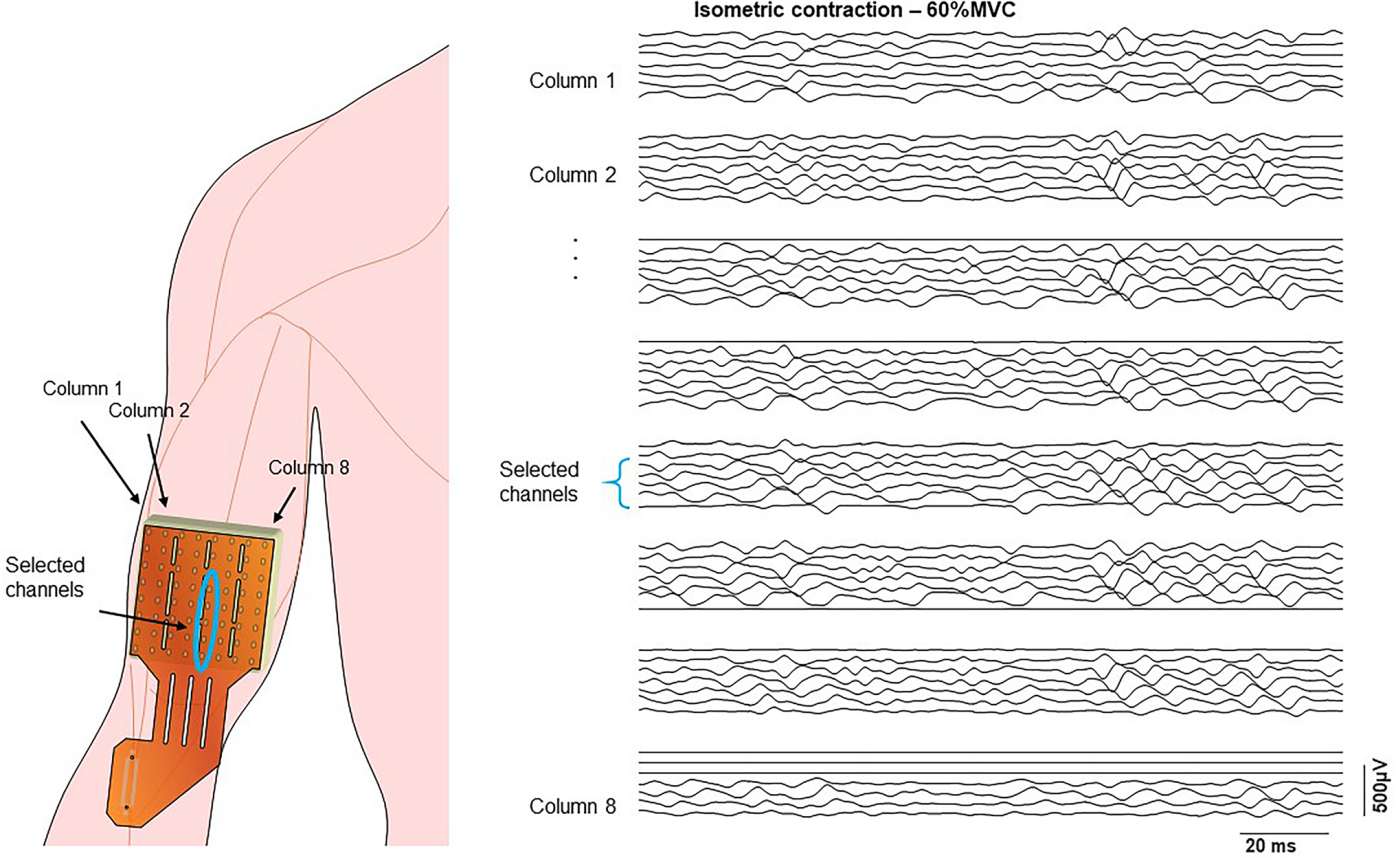
Representation of the position of sEMG array on the biceps brachii muscle. An example of EMG signals detected in single differential mode from each column of a FSHD patient during an isometric elbow flexion at 60% MVC is shown on the right panel. The innervation zone can be identified by the V shape of the signals. The selected channels for muscle fiber estimation are located in the distal portion of column 5, where the pure propagation of motor unit action potentials is visible between the innervation zone and the distal tendon

The channels used for CV estimation were selected based on visual inspection of individual differential signals, along one of the array columns, as described previously (Beretta-Piccoli et al. 2017; Fig. 1), and their number typically ranged from four and seven (Farina et al. 2004). CV was estimated using a multichannel algorithm (Farina and Merletti 2003) on single differential signals based on matching temporally and spatially filtered signals, using non-overlapping signal epochs of 1 s, on the selected channels. Changes in CV during fatiguing contractions have a profound impact on the shape of the motor unit (MU) action potential waveform and therefore on the amplitude and spectral variables extracted from the sEMG signal. Estimation of the CV slope (i.e. rate of change), might be useful to characterize the peripheral components of muscle fatigue during an isometric task (Merletti and Farina 2016) and this variable may be considered as one of the most robust EMG fatigue indices (Kollmitzer et al. 1999; Linssen et al. 1993; Rainoldi et al. 2001; Dedering et al. 2000).

Each of the selected signal epochs was used to estimate the mean frequency of the power spectrum (MNF) and the fractal dimension of the sEMG signal (FD); these variables were averaged over all selected channels. MNF (a parameter used to quantify the changes in the spectral content of the sEMG signal based on the Fourier transform) was computed offline with a numerical algorithm (Merletti et al. 1990) using the following calculation formula (Rampichini et al. 2020):

$$MNF= \frac{{\int }_{f1}^{f2}f\bullet PS(f)\bullet df}{{\int }_{f1}^{f2}PS(f)\bullet df},$$

where PS(f) is the sEMG power spectrum calculated by Fourier transform, and *f*1 and *f*2 determine the bandwidth of the surface electromyography (*f*1 = lowest frequency and *f*2 = highest frequency of the bandwidth). MNF is related to changes in muscle fiber CV and subsequent changes in intracellular action potential duration (Bigland-Ritchie et al. 1981). It has been shown during static contractions that MNF shifts to lower frequencies with increasing fatigue (Lindström et al. 1977; Merletti et al. 1990; Merletti and Lo Conte 1997; Viitasalo and Komi 1977), due to decreased CV as a result of local metabolic changes in the working muscle, mainly H^+^ and K^+^ distribution across the sarcolemma (Dimitrova and Dimitrov 2003; Masuda et al. 1983). However, modifications of MU action potential shape, MU firing rate and synchronization may also contribute to MNF changes (Bigland-Ritchie and Woods 1984; Brody et al. 1991; Gabriel and Kamen 2009).

FD was estimated using the box-counting method as previously reported (Gitter and Czerniecki 1995). Briefly, a grid of square boxes is used to cover the EMG signal, and the number of boxes through which the signal passes is counted. If one decreases the side of the boxes in a dichotomous process, the number of boxes counted increases exponentially. However, if one plots the logarithm of the number of boxes required to cover the signal against the logarithm of the inverse of the box area, one obtains an approximately linear relationship. The slope of the interpolation line (estimated by the least mean squared method) is the FD (Mesin et al. 2009). Therefore, the following expression defines the FD of the sEMG signal:

$${\text{FD }} = \, \log \, N \, / \, \log \, \left( {1/L} \right),$$

where *N* is the number of boxes required to cover the signal, *L* is the box side, and the ratio is the slope of the interpolation line.

FD has been proposed as an index to monitor changes in the sEMG signal during an isometric fatigue task (Beretta-Piccoli et al. 2015; Boccia et al. 2016; Mesin et al. 2009). Although the use of non-linear analysis of the sEMG signal is desirable, as it is more sensitive than spectral analysis for assessing performance fatigability(Farina et al. 2002), it is difficult to relate these parameters to physiological changes in muscle properties resulting from fatigue. Nevertheless, Mesin et al. (2016) demonstrated an inverse relationship between FD and MU synchronization and a positive relationship with the MU firing rate during simulated isometric fatigue contractions.

Performance fatigability was indirectly quantified as the slopes of the considered sEMG variables during endurance contraction.

## Statistical analysis

Descriptive statistics were used to represent the variables included in the comparative analysis; categorical variables were described by frequency distributions, while continuous variables were described by synthetic indicators (median and interquartile range, IQR). At the bivariate level, the analyses were conducted using nonparametric statistical indicators and tests to account for the small sample size and generalized non-normality of the distributions. Linear regression over time was applied to MNF, CV and FD to extract initial values and slopes, the latter normalized with respect to their initial values.

Differences in the sEMG measures between FSHD patients and HC, as well as differences in the continuous clinical variables related to FSHD categories and asymmetry, were assessed using the Mann–Whitney U test. The Cohen’s r estimate of effect size was calculated using (Cohen 1988):

$$r= \frac{\left|z\right|}{\sqrt{N}},$$

where z is the value obtained in the Mann–Whitney U test and N the total number of observations, as suggested by Fritz et al. (2011). Cohen’s guidelines outline that the effect size is low if the value of r varies around 0.1, medium if r varies around 0.3, and large if r varies more than 0.5 (Coolican 2009). Eventually, the Wilcoxon signed-rank test was run to determine differences between predicted and measured maximal voluntary strength in patients with FSHD, and whether the slopes of the EMG parameters at 20% MVC differed from zero. The statistical significance was set at α = 0.05. All statistical analyses have been carried out with Stata/IC 16.0 (StataCorp, College Station, Texas, USA).

## Results

### Socio-demographic and clinical variables

Twelve out of 17 patients belonged to category “A”, according to Ricci et al. (2016), presenting facial and scapular girdle muscle weaknesses. More accentuated muscle weakness on the right side was observed in nine patients, eight of whom were right-handed (Table 1, “Right > Left” category), while it was more accentuated on the left side for three patients and was equally distributed in five of them. The number of patients *per* D4Z4 contraction/number of repeats is described in Table 1. The median clinical score assessing the severity of FSHD was 4 [IQR = 6.25], while the median scapular girdle involvement score was 2 [IQR = 1]. The difference in median age between patients with FSHD and HC was not statistically significant.

### Perceived fatigability

Perceived fatigability, assessed in patients with FSHD using the CIS fatigue subscale, was reported as mild (25.5, [IQR = 15]).

### Performance fatigability

**Fig. 2:**
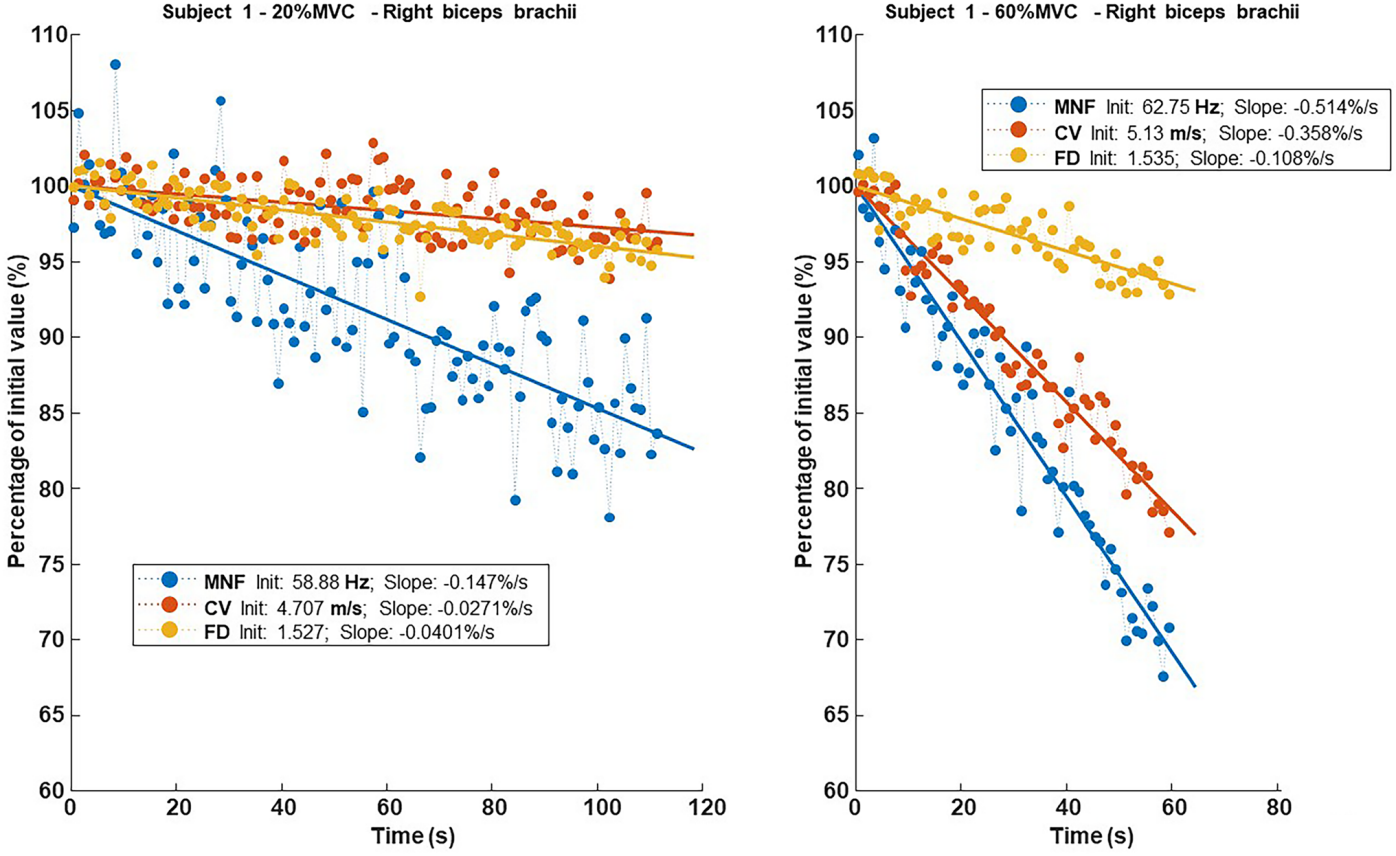
Time course of mean power spectrum frequency (MNF), muscle fiber conduction velocity (CV) and fractal dimension (FD) for a representative patient with FSHD. Surface EMG signals were acquired from the biceps brachii using bi-dimensional arrays during isometric contractions at 20% and 60% MVC. Data are normalized with respect to their initial values

**Fig. 3:**
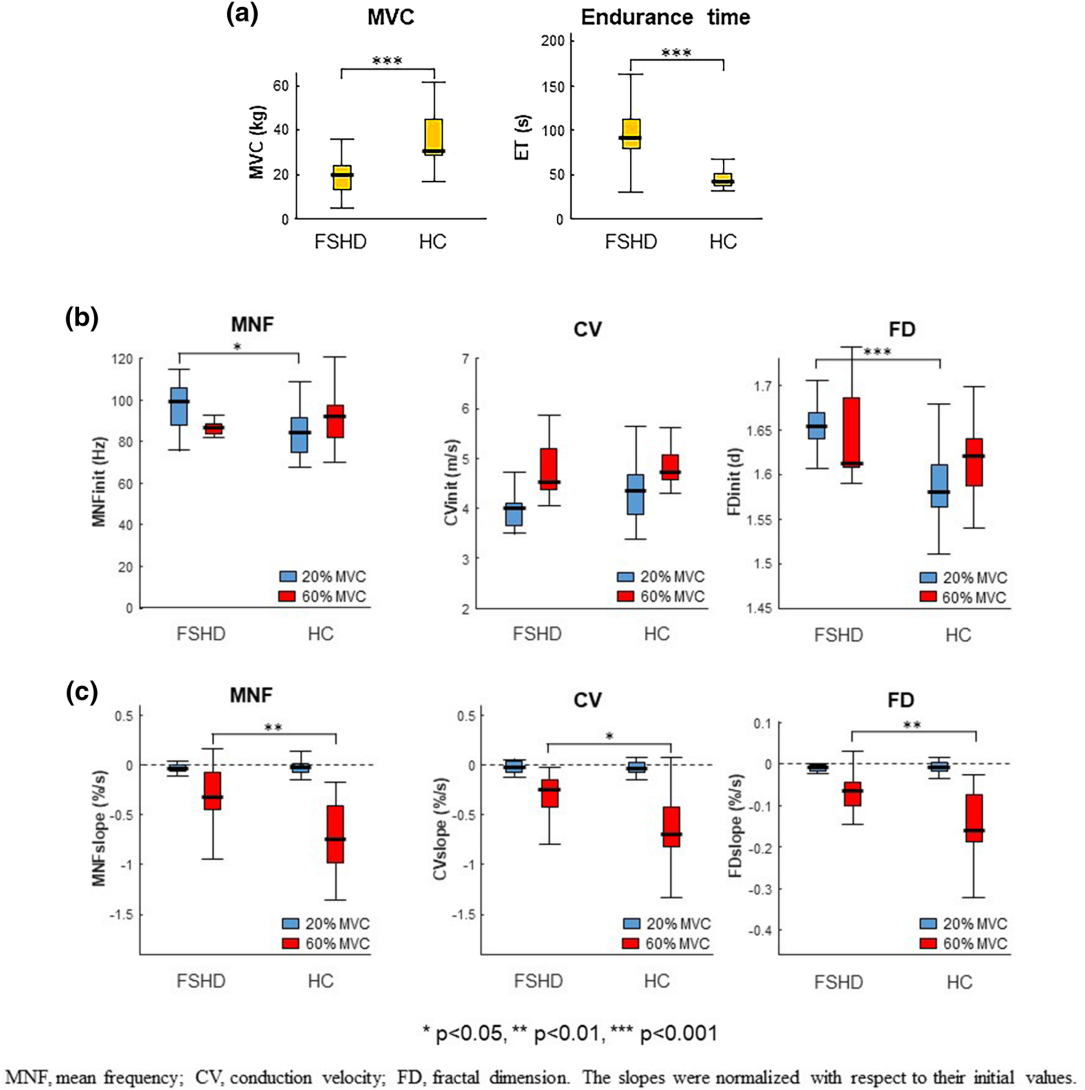
Box-and-whisker plots of **a** maximal voluntary contraction (MVC) of isometric elbow flexions, and endurance time at 60% MVC; **b** initial values and **c** slopes of mean power spectrum frequency (MNF), conduction velocity (CV) and fractal dimension (FD) during the 20% and 60% MVC. Slopes were normalized with respect to their initial values. HC, healthy controls. Asterisks denote statistical significance at * p < 0.05, ** p < 0.01, *** p < 0.005

The time courses of MNF, CV and FD during 20% and 60% MVC are shown in Fig. 2 for a representative subject. The hypothesis test highlighted some statistically significant differences between the two groups, which are shown in Fig. 3: (1) the initial values of MNF and FD at 20% MVC yielded higher values in the FSHD group (*p* 0.05 and p 0.001, respectively), while their negative slopes showed a less steep decline at 60% MVC (*p* 0.01). (2) A similar decrease was also observed for the negative slope of CV at 60% MVC in the FSHD group (*p* 0.05). At 20% MVC, the slope of MNF was not different from 0 in the FSHD group (*p* = 0.14), and in both groups for CV (*p* = 0.08 and *p* = 0.53, respectively). In addition, exerted force was lower in the FSHD group, while endurance time was longer compared to the HC group (*p* = 0.001). Effect size analysis revealed high values for MNF slope and FD slope during 60% MVC contraction (r estimates and 0.56 and 0.51, respectively), FD initial value during low-level contraction (*r* = 0.61), and maximum voluntary force (*r* = 0.62) and endurance time (*r* = 0.63). Moreover, the initial values of MNF at 20% MVC and the slope of CV at 60% MVC showed a medium effect (r-estimates and 0.33 and 0.37, respectively). A smaller effect size was found for the remaining parameters.

### Muscle weakness in FSHD patients

The median MVC at 60° of isometric elbow flexion was 19.79 [IQR = 10.87] kg, while the predicted MVC in heathy subjects was 28.82 [IQR = 11.76] kg. Consequently, the maximal strength was 31.3% significantly lower than the predicted value (*p* < 0.01).

### Differences in clinical variables related to FSHD category and asymmetry

Because of the small sample size, for a robust assessment of differences in clinical variables, FSHD category and asymmetry were recoded into binary variables: “A” and “Not A”, and “Right > Left” and “Not Right > Left” for FSHD categories and asymmetry, respectively. D4Z4 contraction length showed no statistically significant differences between FSHD categories, whereas patients with more severe right-sided muscle (category “Right > Left”) reported a significantly lower FSHD severity (*z* = − 2.156; *p* < 0.05).

## Discussion

In this study, sEMG parameters, known as indirect indices of performance fatigability, were examined in FSHD subjects and HC during isometric fatigue contractions of the elbow flexors. The results showed significant differences in all parameters considered between the two groups; in particular, FSHD patients presented lower MVC and lower levels of performance fatigability (i.e. reduced slope of MNF, CV and FD), and increased time to exhaustion during isometric fatigue contraction at 60% MVC.

### Muscle weakness, strength, and endurance time

In agreement with previous studies, the results presented a fairly consistent picture, characterized by the inability of patients with FSHD to exert maximal strength comparable to the one of HC (Bachasson et al. 2014; Doix et al. 2017; Kalkman et al. 2007; Turki et al. 2012). This finding is corroborated by an increased predicted muscle weakness experienced by patients with FSHD. A recent study by Lassche et al. (2020) hypothesized that muscle weakness in FSHD is not caused by alterations in sarcomeric contractility, or excitation–contraction coupling (Vandebrouck et al. 2002), but is due to DUX4-induced toxicity and consequential loss of muscle fibers or to an unknown impaired metabolic cause.

In addition, the patients’ endurance time was significantly longer than in HC, suggesting an increased resistance to the development of fatigue (Fig. 3a), probably for several reasons:

First, patients with FSHD may exhibit fibrosis and fatty infiltration (Friedman et al. 2012) which are associated with changes in muscle composition and, particularly from fast-glycolytic (type IIX) to slow-oxidative (type I) muscle fibers (Celegato et al. 2006). Indeed, these structural alterations evaluated by MRI, have been associated with a significantly decreased phosphocreatine/ATP ratio (Janssen et al. 2014), histological findings of biopsies showing more dominant type I fibers (Dubowitz et al. 2020), and loss of muscle strength. Second, weaker participants have been less fatigable than stronger ones (Hunter and Enoka 2001) because intramuscular pressure is lower, the blood occlusion is also lower (Zwarts and Arendt-Nielsen 1988) and negative feedback from afferent groups III and IV is reduced (Gandevia 2001). Reduced fatigability in patients with FSHD with respect to HC was also described in Doix et al. (2017), before and after short-term neuromuscular electrical stimulation training of the anterior tibialis muscle, which may be affected similarly to the BB in earlier stages of the disease (Olsen et al. 2006; Dorobek et al. 2013).

### Performance fatigability

Interestingly, the fatigability indices extracted from the sEMG signal suggested an increased resistance to fatigue in the FSHD patients (Fig. 3b). Indeed, the reduction in the normalized slope of MNF, CV and FD during high-level fatigue contraction, a sign of an increase in performance fatigability (Merletti and Farina 2016; Beretta-Piccoli et al. 2017 and 2020), was significantly lower in patients with FSHD compared to HC. Similar results were reported by Schillings et al. (2007), who described lower peripheral fatigue in FSHD patients compared to controls, which was directly related to lower maximal effort and blood flow occlusion compared to controls. Moreover, the lower decrease in CV slope may also be explained by a larger contribution of fatigue-resistant type I muscle fibers, a well-known feature of FSHD (D'Antona et al. 2007).

However, the reduction in fatigability also affects the slope of MNF (which is related to both a reduction in muscle fibers CV and an increase in MU firing rate and synchronization Bigland-Ritchie and Woods 1984; Brody et al. 1991; Gabriel and Kamen 2009), and the slope of FD (which is related to an increase in MU firing rate and synchronization (Rampichini et al. 2020). It is, therefore, reasonable to hypothesize that the reduced fatigability in FSHD is also due to central factors, as also suggested by Schillings et al. (2007).

On the contrary, during the 20% MVC contraction, the normalized slopes of the fatigability indices did not differ, suggesting that the level of contraction was not fatiguing (confirmed by the slopes of CV, which did not differ from 0 in the two groups) and hypothesizing that there is a greater recruitment of type I muscle fibers than type II in FSHD patients (Fig. 3b).

### Initial values

During low-level contraction, which was non fatiguing for both groups, increased initial values of MNF and FD were observed in patients with FSHD (Fig. 3c). This result is not surprising, since a greater impact of the physiological factors affect the initial values of MNF. Unexpectedly, no significant difference was observed in muscle fiber CV, although reduced CV levels have been reported in previous studies in other neuromuscular disorders (Naumann and Reiners 1996; Al-Ani et al. 2001; Butugan et al. 2014).

Probably, this is related to a heterogeneous control group, in which individuals have different fatigue patterns. On the contrary, the result of the initial values of FD of the sEMG signal, which quantitatively indicates the chaotic behavior of the signal, and is related to the degree of interference (Mesin et al. 2009), suggests an increased complexity of the signal compared to the controls, especially at 20% MVC. Several studies determined that FD can be used to quantify the complexity of MU recruitment patterns (Arjunan and Kumar 2016). Since it is well known that MU recruitment is impaired in neuromuscular disorders and more MUs are recruited in myopathies even at low forces (Sanders et al. 1996), an increase in signal complexity may be hypothesized. Furthermore, Derry et al. (2012) observed a more complex MU action potential morphology during low-level (10–20% MVC) isometric contractions of the BB in FSHD patients compared to controls. Eventually, they showed that as the disease progressed and muscle fibers were increasingly lost due to the degenerative process, complexity increased.

### Influence of FSHD category and asymmetry over fatigability

Surprisingly, no statistically significant differences in clinical variables were detected between FSHD categories classified as A or not-A, suggesting that the two groups are comparable from a clinical point of view. In particular, the fact that the D4Z4 array does not differ between the categories seems to underline a labile relationship with the clinical picture and even that the size of the deletion is not sufficient to tell whether a patient with eight repeated units belongs to A or not-A category. The two categories considered share common pathophysiological traits, at least as far as fatigue is concerned. For instance, the most conclusive common event is loss of strength and a fast-to-slow shift of muscle fiber type composition.

The results regarding asymmetry, which is a very common feature in FSHD, unexpectedly showed that patients with more accentuated muscle weakness on the right side have a better clinical picture, highlighted by a lower median severity score compared to those with more left sided or equal right/left involvement.

The prevalence of right-sided involvement is consistent with previous findings and has been associated with mechanical factors and, in particular, with preferential use of the right side by right-handed individuals (Brouwer et al. 1992; Tasca et al. 2014).

### Limitations

A limitation of this study is related to the model used to fit time-dependent changes in EMG parameters during the high-level isometric contractions. Linear trend analysis was used. However, changes in MNF and CV may also follow an exponential trend during sub-maximal contractions (Merletti et al., 1991). Here, a linear model greatly simplified the analysis, and inspection of the data showed that the assumption of linear trends was reasonable for all parameters. Moreover, as fatigability assessment is task dependent (Enoka 1995), protocol specifications are known to influence findings and underlying mechanisms of fatigue. Furthermore, the majority of studies on fatigability in FSHD patients have been conducted using electrical stimulation, thus the results may not be comparable. In addition, we evaluated fatigability in the dominant BB only, which may not represent the disease condition of the whole individual. Although the BB is often one of the first muscles to manifest signs of weakness in FSHD, it is not excluded that muscles of the shoulder girdle with a more complex architecture (e.g. the trapezius) may be a better choice for evaluating early structural changes. Then, we cannot exclude phenomena of compensation of muscle groups that may affect the developed force. Moreover, our results are based on a small group of patients, thus the statistical power is rather low, especially for the comparisons between patients. Finally, the rate of perceived exertion after the tasks was not measured, so it was not possible to perform a correlation analysis between the state level of perceived and performance fatigability.

## Conclusions

In summary, we reported impaired neuromuscular function in FSHD compared to HC, due to muscle weakness, which caused patients to exert lower MVC and reduced fatigability, as evidenced by longer endurance time and a lower decrease in the slopes of all sEMG fatigability indices considered during sustained isometric elbow flexions.

Further studies need to be conducted to evaluate performance fatigability in the FSHD subcategories and also to investigate the patients’ fatigue induced by functional exercise unrelated to individual MVC (e.g. walking, sit-to-stand transfer) to clarify the impact of fatigue on their activities of daily living.

## Data Availability

Under motivated request to GDA.

## Abbreviations

FSHD: Facioscapulohumeral muscular dystrophy
EMG: Electromyography
MVC: Maximal voluntary contraction
CIS: Checklist individual strength
CV: Conduction velocity
MNF: Mean power frequency
FD: Fractal dimension
IQR: Interquartile range
